# Surface electromyography as a non-invasive method for detecting stress-induced changes in gastrointestinal motility in pigs

**DOI:** 10.1371/journal.pone.0358481

**Published:** 2026-09-15

**Authors:** Orsolya Csötönyi, Gabriella Béres, Ágnes Baráth, László Sarkadi, Zsuzsanna Szőke, Zsófia Molnár, Patrik Plank, Veronika Halas

**Affiliations:** 1 Department of Farm Animal Nutrition, Hungarian University of Agriculture and Life Sciences, (MATE) Kaposvár, Hungary; 2 Department of Animal Biotechnology, Institute of Genetics and Biotechnology, Hungarian University of Agriculture and Life Sciences, Gödöllő, Hungary; University of Life Sciences in Lublin, POLAND

## Abstract

Surface electromyography of the gastrointestinal tract is a non-invasive method for monitoring smooth muscle myoelectrical activity and provides an integrated assessment of gastrointestinal motility. Although prolonged or marked stress is known to impair digestive efficiency in livestock, partly via cortisol-mediated modulation of gastrointestinal smooth muscle activity, it remains unclear whether a single mild stressor can induce detectable alterations in motility. Therefore, this study aimed to determine whether a single mild, experimentally induced stressor alters gastrointestinal motility in pigs and whether surface electromyographic measurements are sufficiently sensitive to detect these changes. Four growing pigs were examined using a within-individual experimental design on a baseline control day and on a treatment day involving intravenous administration of adrenocorticotropic hormone (10 micrograms per kilogram body weight) to induce a mild stress response. Gastrointestinal smooth muscle electrical activity was recorded non-invasively using surface electromyography, and repeated blood samples were collected to determine serum cortisol concentrations over time. Adrenocorticotropic hormone administration produced a clear, time-dependent increase in serum cortisol concentrations, confirming the effectiveness of the experimental stress model. This endocrine response was accompanied by a significant reduction in small intestinal smooth muscle activity, whereas gastric and large intestinal activity showed no statistically significant changes, although a consistent tendency toward reduced motility was observed under stress. These findings indicate that small intestinal myoelectrical activity is particularly sensitive to mild endocrine stress and demonstrate that non-invasive surface electromyography can detect stress-related alterations in gastrointestinal function. This approach may provide a useful tool for investigating stress physiology and monitoring digestive function in livestock.

## Introduction

Electromyography (EMG) refers collectively to methods used to measure muscle activity, while the non-invasive measurement of muscle function is termed surface electromyography (sEMG). Different forms of EMG allow the investigation of both skeletal and smooth muscle function. A specialized variant, smooth muscle electromyography of the gastrointestinal tract (SMEMG), is used to monitor the myoelectrical activity of various segments of the digestive system. This method provides an integrated overview of gastrointestinal motility under both physiological and altered conditions and can be applied to assess gastric, small intestinal, and large intestinal smooth muscle activity [3].

The physiological basis of electromyographic measurements is that when a smooth muscle cell membrane receives a stimulus, the voltage difference between the intracellular and extracellular compartments – the resting membrane potential – changes transiently. The stimulus increases membrane permeability to sodium ions (Na⁺), which enter the cell in large quantities. As a result, the intracellular membrane surface becomes temporarily positive while the extracellular surface becomes negative, a process known as depolarization. Depolarization triggers electrical activation that leads to the generation of an action potential. Shortly afterward, the membrane potential returns to its resting state (repolarization). The electrical activation promotes substantial intracellular release of calcium ions (Ca^2+^), which, after a brief delay, induces smooth muscle fiber contraction [1].

The smooth muscle tissues of the gastrointestinal organs contain intrinsic pacemaker cells known as interstitial cells of Cajal. These cells play a key role in generating and propagating the electrical signals that underlie smooth muscle contractions. They produce characteristic slow-wave electrical impulses, which can be detected through the electrical and mechanical activity of smooth muscle cells, and their frequency can be quantified [2]. Based on these frequency domains – and with appropriate signal-processing techniques – smooth muscle activity can be distinguished from signals originating from skeletal muscle. Reliable software-based analysis therefore requires prior knowledge of the characteristic frequency ranges of each intestinal segment. Currently, sEMG has not become an established method for analyzing gastrointestinal motility in livestock species, including pigs. Only a very limited number of studies have applied SMEMG in pigs [3,4], and none have examined gastrointestinal myoelectrical activity under stress conditions. Although the stress-induced reduction of gastrointestinal motility has been documented across several species [5–7], the available literature indicates that these effects have not yet been supported by quantitative evidence using non-invasive monitoring approaches. Furthermore, a fully standardized protocol for measurements in awake, non-anesthetized large animals has not yet been established. In rats, characteristic cycles-per-minute (CPM, defined as the number of complete myoelectrical contraction cycles recorded per minute) ranges for distinct gastrointestinal segments were defined under controlled experimental conditions in which the organs were examined separately to avoid potential cross-interference [8]. In pigs, Nagy et al. demonstrated the applicability of rat-derived CPM ranges [3], and Salimi-Jazi et al. [4] reported segmental CPM values obtained *in vivo* from awake animals using simultaneous internal and external recordings [4]. However, *in vivo* measurements inherently allow physiological interactions between adjacent gastrointestinal segments, which may influence segment-specific myoelectric patterns. Therefore, until more extensively validated and harmonized porcine reference data become available, the use of CPM ranges derived from experimentally isolated segments may represent a pragmatic and methodologically consistent approach in pigs.

Importantly, while the establishment of universally accepted reference CPM ranges in pigs remains under refinement, the methodological feasibility of *in vivo* measurements has been convincingly demonstrated. Recently, Salimi-Jazi et al. compared the measurements obtained from surgically implanted internal electrodes with those recorded using surface electrodes in the gastrointestinal organs (stomach, small intestine, and large intestine) of Yucatan minipigs [4]. The results demonstrated a high correlation between the two methods, supporting the potential applicability of non-invasive measurements. They also reported that non-invasive, continuous monitoring of gastrointestinal myoelectrical activity over several days can serve as a useful tool for diagnosing motility disorders. This implies that stress-induced alterations in gastrointestinal activity may also be detectable using such measurement techniques.

The aim of the study was to determine whether a single mild, experimentally induced stressor alters gastrointestinal motility in pigs and whether surface electromyographic measurements are sufficiently sensitive to detect these changes.

## Materials and methods

The animal experiment has been approved by the Animal Welfare Committee and Scientific Ethics Council for Animal Experiments (Somogy County District Office of Kaposvár Food Chain Safety and Animal Health Unit; Ethical approval number: SO/31/00881–2/2023 (KA-3926)).

At the end of the study, the animals were not euthanized. Following completion of the experimental procedures, all pigs underwent veterinary examination, and after confirmation of their healthy clinical status, they were returned to the food production chain in accordance with the approved animal welfare protocol.

The experiment was conducted in December 2023 at the Hungarian University of Agriculture and Life Sciences, Department of Farm Animal Nutrition. Four Topigs × Duroc growing barrows (25.9 ± 0,62 kg body weight) were housed individually in concrete-floored pens without bedding and were fed twice daily (08:00 and 15:00 h) with *ad libitum* access to drinking water.

### Preparation and habituation of the animals

**Fig 1:**
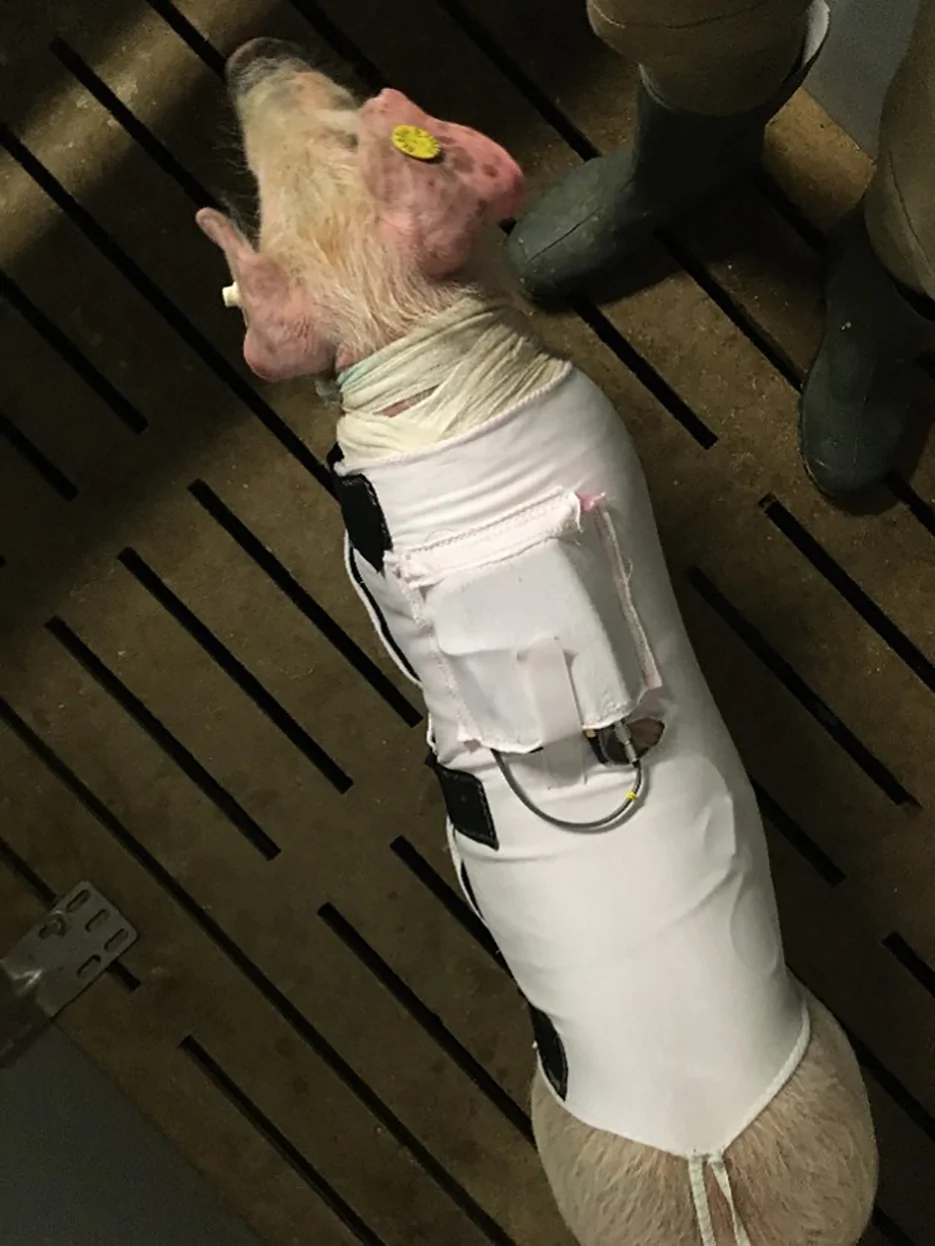
One of the experimental pigs with vest and Holter device.

Because the study focused on the effects of stress induction within a self-control design, animals underwent a one-month habituation period to ensure tolerance of human presence, handling, and wearing a vest, which held the wires and the Holter device in position for sEMG measurements (Fig 1.).

To enable repeated blood sampling (16 samples/day) and intravenous ACTH administration **while minimizing procedure-related stress**, permanent jugular venous catheters were surgically implanted 4 days before the experiment. General anesthesia was induced with intramuscular Zoletil® 50 (4 mg/kg), followed by inhalational anesthesia with isoflurane in oxygen. After induction, the animals were intubated, and anesthesia was maintained with 2% isoflurane in oxygen. The catheter was inserted into the jugular vein through a small skin incision and secured with sutures. Local analgesia was provided by infiltration and topical application of 1% lidocaine prior to intubation and venous catheterization. Physiological parameters, including electrocardiogram (ECG), oxygen saturation, and body temperature, were continuously monitored throughout surgery. Following surgery, the incision site and sutures were inspected daily and cleaned as needed throughout the experimental period. Catheter patency was maintained by daily flushing with heparinized saline. All procedures were performed by appropriately trained personnel in accordance with the approved animal welfare protocol.

### Study design and stress induction

**Fig 2:**
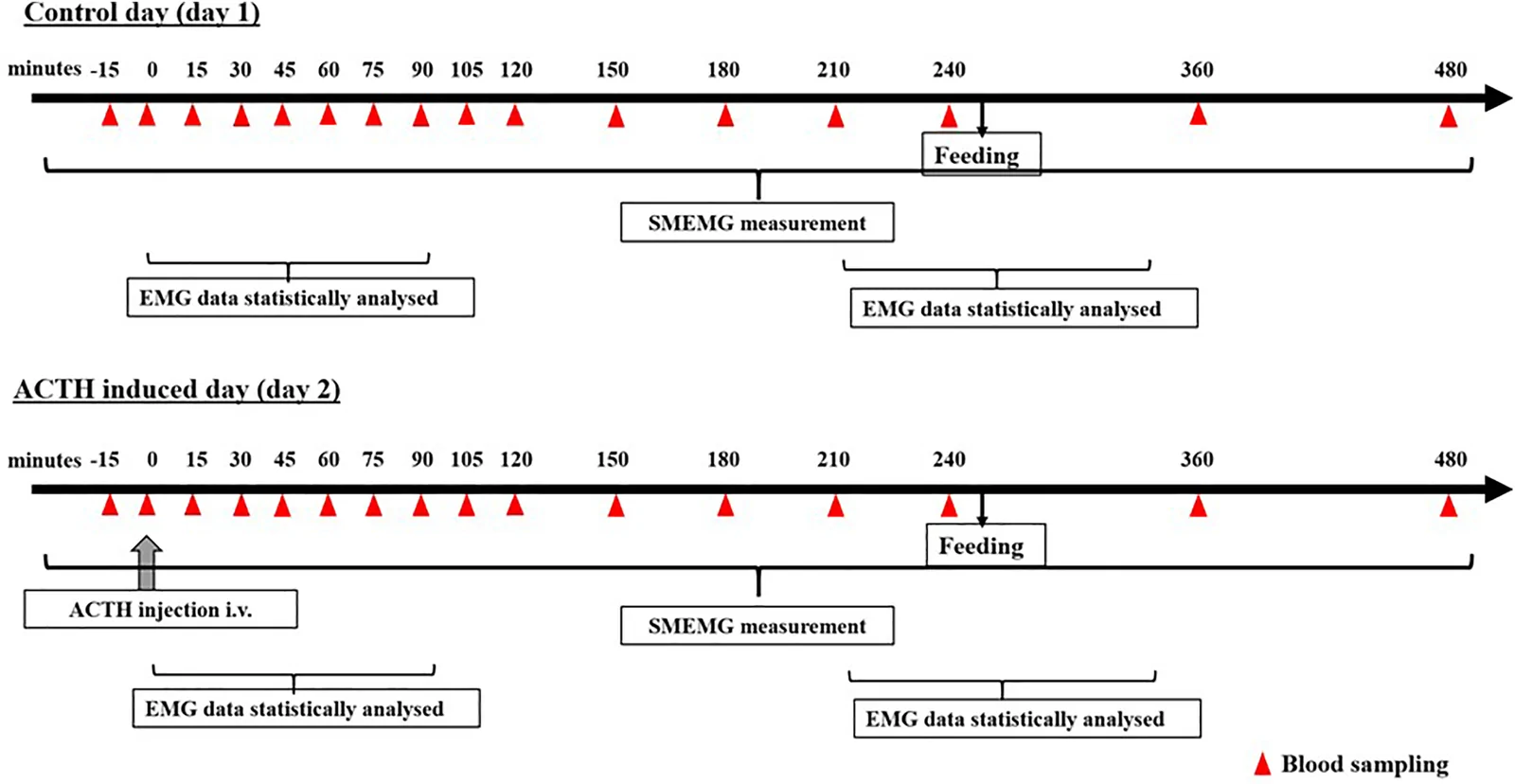
Study design and sampling schedule on experimental days.

Each pig was evaluated on two consecutive days (Fig 2.): a control day without stress induction, and a treatment day during which stress was elicited via intravenous administration of ACTH (Synacthen; 0.25 mg/mL) through permanent venous catheter at a dose of 10 µg/kg body weight, representing a mild, short-term, cortisol-mediated stress stimulus.

### Blood sampling and serum cortisol measurements

At each sampling time point (Fig 2.), 4 mL of blood was collected into serum tubes, centrifuged at 2,500 × g for 15 min at 4 °C, and the obtained serum was stored at −20 °C until analysis. Serum cortisol concentrations were quantified using a quantitative enzyme-linked immunosorbent assay (ELISA) kit (Cortisol ELISA, Gold Standard Diagnostics, Product No.: DNOV001, Lot: CORT-6028A) following the manufacturer’s protocol. No extraction or dilution steps were necessary prior to the analysis. Briefly, 20 µL of standards and samples were dispensed into the wells, accompanied by 200 µL of enzyme conjugate, and incubated for 1 h at 37 °C. Following this, the wells were washed with 300 µL of wash solution per well and 100 µL of TMB substrate was added. The plates were then incubated for 15 min at room temperature in the dark. The enzymatic reaction was terminated by adding 100 µL of stop solution, and the optical density was measured at 450 nm using a Multiscan ELISA reader (Waltham, MA, USA).

### Surface electromyographic measurements and data analysis

**Fig 3:**
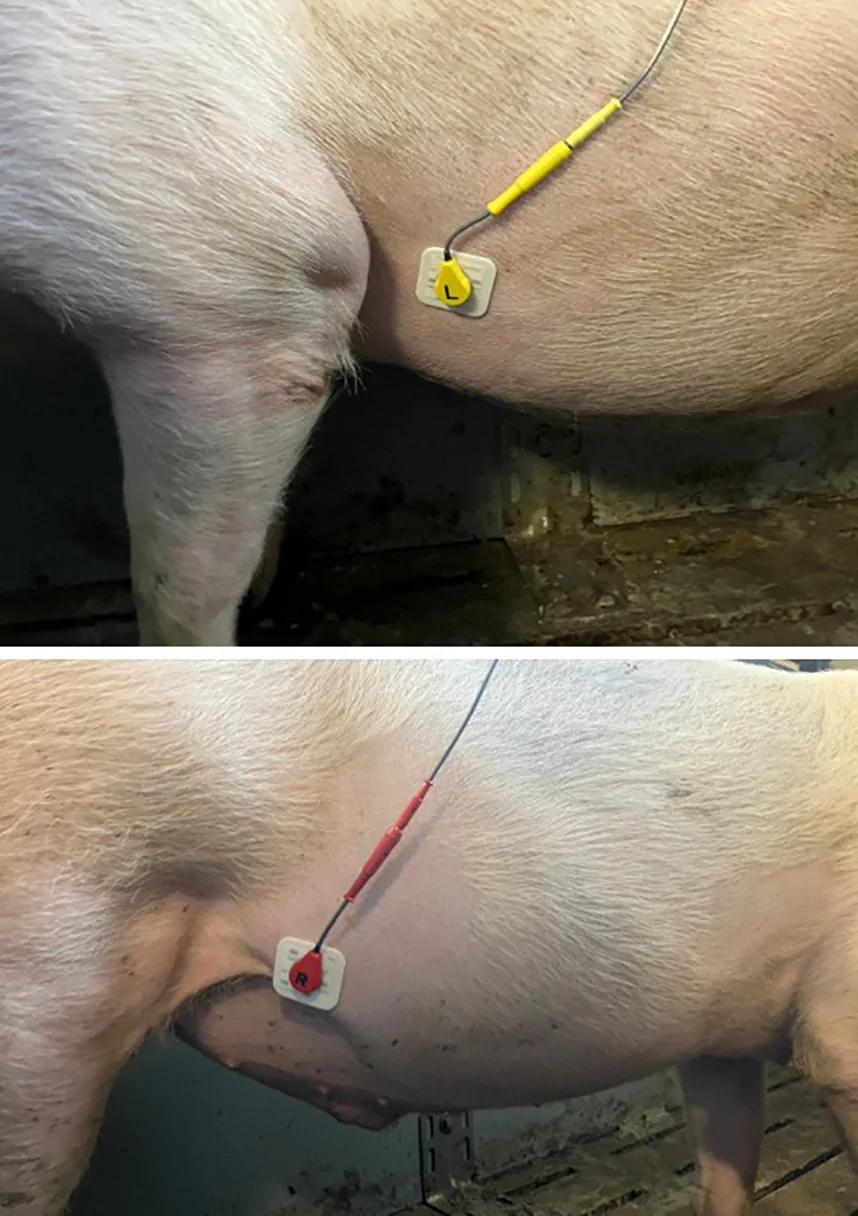
Electrode placement during EMG measurements.

SMEMG data were collected using a Holter device (DR4CH01, MSB-Met Kft., Hungary) connected to two disposable, self-adhesive foam surface electrodes (3M™ Red Dot™, 4 × 3.3 cm; 3M, St. Paul, USA) placed after shaving the area and cleaning the skin with 96% (v/v) ethanol. One electrode was positioned on the left side near the heart, right behind the front left leg. The other electrode on the right side was placed on the fold of skin connecting the pig’s thigh and lower abdomen (Fig 3.). Measurements were recorded for 9 hours (Fig 2.), of which 8 hours were analyzed using EasyChart software. The recording was segmented into 30-minute intervals. Two predefined analysis windows were applied: from the time of ACTH administration to 2 hours post-injection, and from 30 minutes before feeding to 90 minutes after feeding (in the afternoon). Feeding occurred 4 hours after ACTH administration.

The electrical signals were recorded using the Holter recording system, which included built-in hardware signal conditioning. The gastrointestinal electrical activity channels were amplified with a gain of ×2000 and recorded with a bandwidth of 0.01–1 Hz. Signals were digitized at a sampling rate of 1000 samples/s (1000 Hz). No additional preprocessing or artifact-reduction procedures were applied by the Holter system prior to signal analysis.

**Fig 4:**
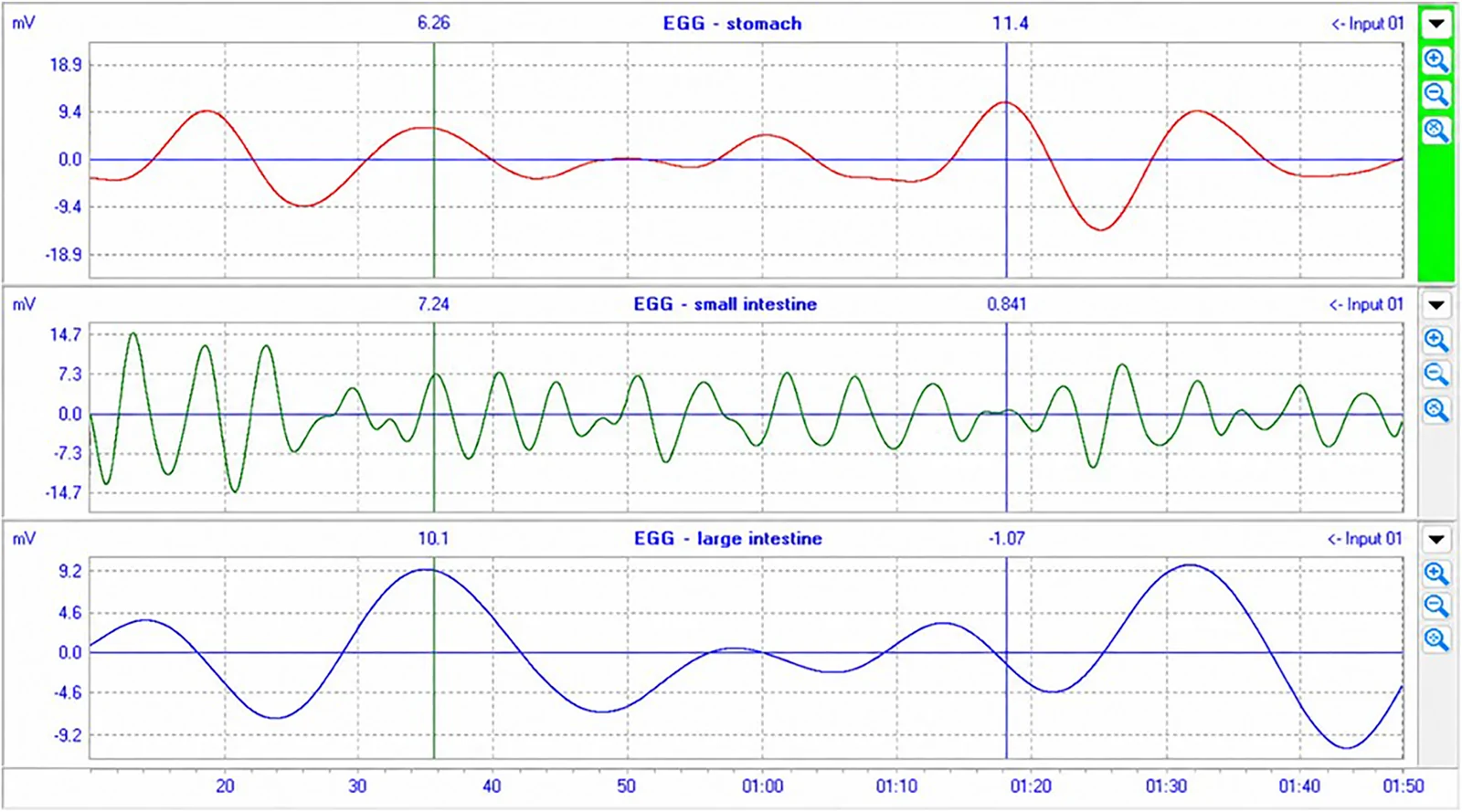
Example of raw gastrointestinal myoelectrical signals from the stomach, small intestine, and large intestine displayed in the software before FFT analysis; time is shown on the x-axis.

Organ-specific signal separation was performed using predefined frequency filters implemented in the EasyChart software (Fig 4). The applied frequency ranges were given in CPM (cycle-per-minute) defined as the number of complete myoelectrical contraction cycles recorded per minute and set 3–5 CPM for the stomach, 10–20 CPM for the small intestine and 1–3 CPM for the large intestine. The frequency unit CPM is conventionally used for the characterization of gastrointestinal myoelectrical activity; for comparison with SI frequency units, CPM values can be converted to Hertz (Hz; 1 Hz = 60 cycles/min).

Following frequency filtering, Fast Fourier Transform (FFT) analysis was performed within each predefined organ-specific frequency range, and the Power Spectrum maximum (PSmax) value was determined for each 30-minute interval. PSmax was defined as the maximum value of the power spectrum curve obtained from FFT analysis, representing the highest spectral power detected within the selected frequency range. Gastrointestinal myoelectrical activity was quantified based on these PSmax values.

### Statistical analysis

Due to the small sample size (n = 4), formal tests of normality have inherently low statistical power and limited ability to reliably detect deviations from normality. Accordingly, normality assessment was not based solely on hypothesis testing; the results of the Shapiro–Wilk test were interpreted in conjunction with graphical diagnostics, including Q–Q plots and boxplots, to evaluate distributional assumptions more comprehensively. Treatment effects and temporal dynamics were evaluated using linear mixed-effects models (LMMs) fitted with the *lme4* and *lmerTest* packages in R (version 4.5.2). The model structure was specified as:


$$\begin{array}{c} Result\sim Treatment\times Time+(\text{1}|pig), \end{array}$$

where *pig* was included as a random intercept to account for repeated measurements within individuals. Fixed effects were tested using Type III analysis of variance with Satterthwaite’s approximation for degrees of freedom. *Post hoc* pairwise comparisons were conducted using the *emmeans* package with Tukey’s adjustment for multiple testing. Results are presented as mean ± standard error of the mean (SEM), and a significance level of P < 0.05 was applied throughout.

The R scripts used for statistical analyses are publicly available at https://doi.org/10.5281/zenodo.21720478.

## Results & discussion

### Serum cortisol levels during the experimental days

**Fig 5:**
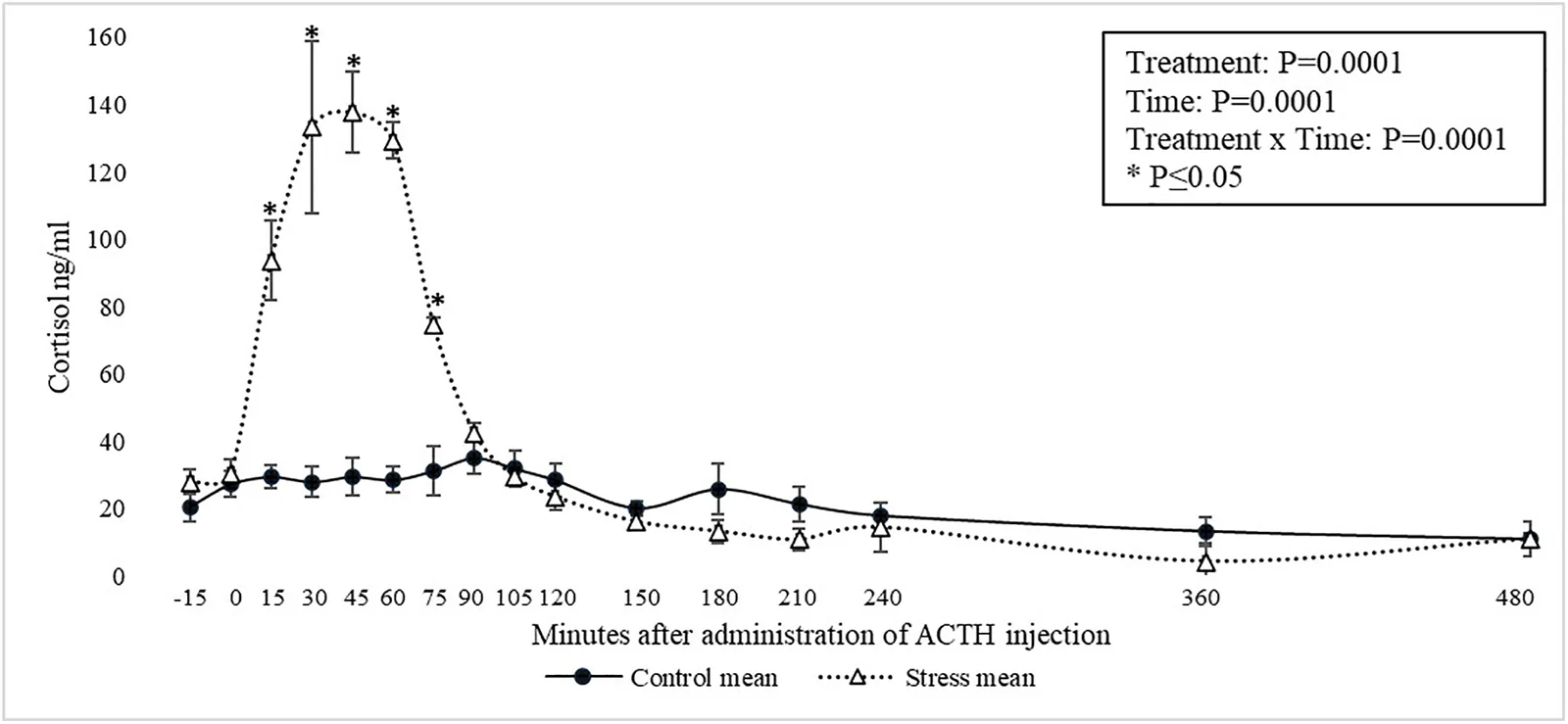
Blood serum cortisol concentrations (mean ± SEM) on control and ACTH-injected days.

The linear mixed-effects model revealed significant main effects of Treatment (control vs. stress; P < 0.0001), Time (P < 0.0001), and a significant Treatment × Time interaction (P < 0.0001). *Post hoc* Tukey-adjusted pairwise comparisons at each sampling time showed that cortisol levels were significantly higher under the stress condition compared to control specifically at 15–75 minutes post ACTH injection (all P < 0.001). At the remaining time points (0–15 min and 90–480 min), the differences were not statistically significant (all P > 0.05) (Fig 5.).

During the control day, cortisol concentrations remained relatively stable, staying within the normal physiological range [9]. On the stress day, a sharp increase in cortisol was observed following ACTH injection, with peak concentrations occurring during the mid-phase (15–75 min) and gradually declining toward baseline thereafter. This pattern aligns with the expected pharmacodynamic response for Synacthen® (Novartis), confirming the effectiveness of the stimulation protocol. Similar kinetics have been documented in porcine models, where synthetic ACTH administration (10 μg/kg) elicited peak plasma cortisol within 60–70 minutes and returned to baseline within 180–240 minutes [10].

The minimal variation in cortisol on the control day suggests that the animals tolerated the procedures well, likely due to the preceding habituation period. In contrast, the relatively large standard errors on the stress day indicate substantial inter-individual variability in cortisol responses, consistent with previous reports in pigs [11,12] and humans under standardized stress protocols [13,14]. These findings support the robustness of the ACTH-induced stress model while highlighting individual differences in stress sensitivity.

### Gastric smooth muscle activity

**Fig 6:**
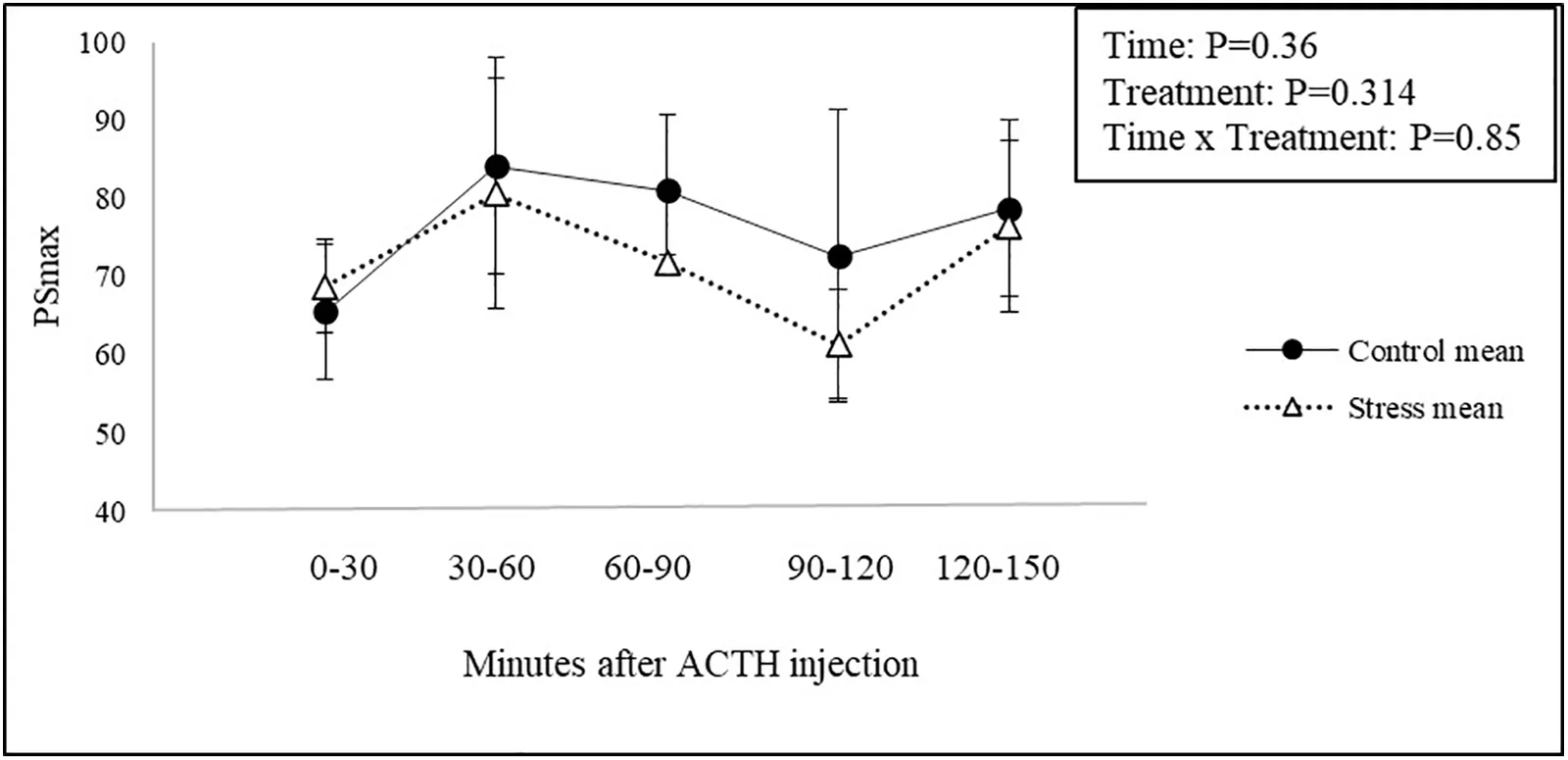
Gastric PSmax values (mean ± SEM) on control and ACTH-injected days, 0-150 minutes after ACTH injection.

The linear mixed-effects model revealed no significant main effect of Treatment (control vs. stress; P = 0.314), Time (P = 0.36), and Time × Treatment interaction (P = 0.85). This indicates that gastric smooth muscle activity was not interfeared by the induced stress either during the cortisol peak or thereafter (Fig 6.).

Although the applied ACTH-induced stress protocol successfully elevated serum cortisol levels, no statistically significant changes in gastric smooth muscle activity were observed. Visually and numerically, a modest reduction in gastric activity was apparent under the stress condition, but this decrease did not reach statistical significance. This finding is consistent with previous reports indicating that stress-induced gastrointestinal motility changes are highly context-dependent, influenced by the type, intensity, and duration of the stressor, as well as the species and experimental conditions [6,15,16]. In particular, mild, short-term endocrine stressors may not be sufficient to induce immediate or pronounced changes in gastric motility. Under the present experimental conditions, the data suggest that gastric smooth muscle activity exhibits relative resistance to mild ACTH-induced stress.

**Fig 7:**
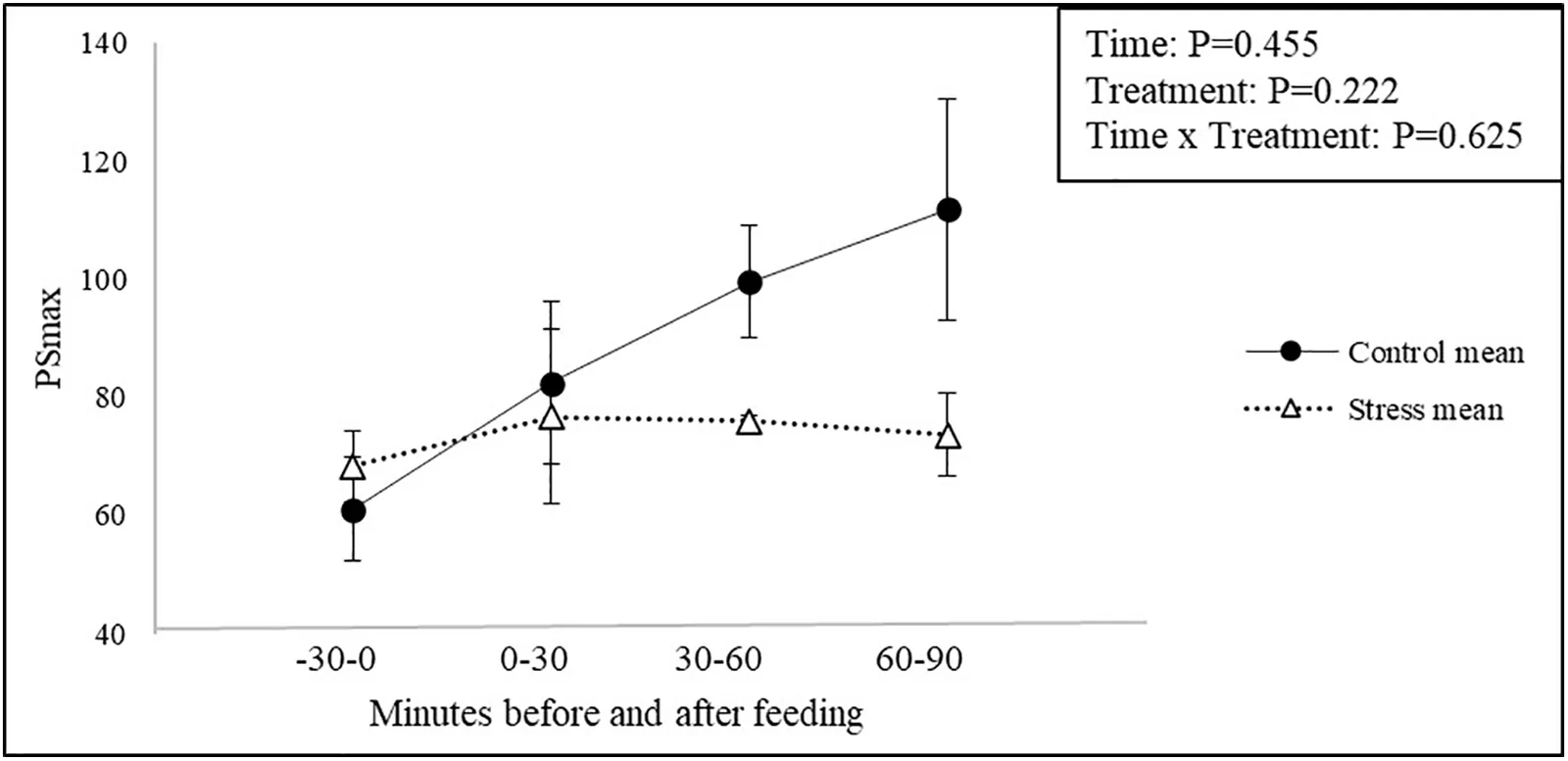
Gastric PSmax values (mean ± SEM) on control and ACTH-injected days, 30 minutes before and 90 minutes after feeding.

To assess the potential residual effects of ACTH-induced stress on feeding-associated motility responses, animals were fed 4 hours after ACTH administration, and smooth muscle activity was recorded from 30 minutes before to 90 minutes after feeding. The linear mixed-effects model revealed no significant main effect of Treatment (control vs. stress; P = 0.222) on gastric smooth muscle activity during the afternoon feeding period. No significant main effect of Time was detected (P = 0.455), and no significant Treatment × Time interaction was observed (P = 0.625) across the 30-minute pre-feeding interval and the 90-minute post-feeding period (Fig 7.).

Nevertheless, on the control day, animals exhibited the expected postprandial increase in gastric motility, whereas this physiological rise appeared attenuated in the stress condition. Although no statistically significant Treatment, Time, or Treatment × Time effects were detected, the observed descriptive pattern may justify further investigation in larger cohorts. It is well established that stress can disrupt gastrointestinal motility through activation of specific brain nuclei and neuronal pathways, accompanied by the release of central and peripheral neurotransmitters. Substantial neuroanatomical and functional evidence from animal studies indicates that corticotropin-releasing factor plays a central role in mediating stress-induced alterations of gut motor function [6]. Although Williams et al. [17] did not observe delayed gastric transit following mild stress induction [17], the absence of a significant effect in the present study likely reflects the mild intensity and short duration of the ACTH-induced stress, as well as the animals’ prior habituation to human presence, handling, and experimental procedures, which can attenuate stress effects at the level of visceral smooth muscle [18].

### Small intestinal smooth muscle activity

**Fig 8:**
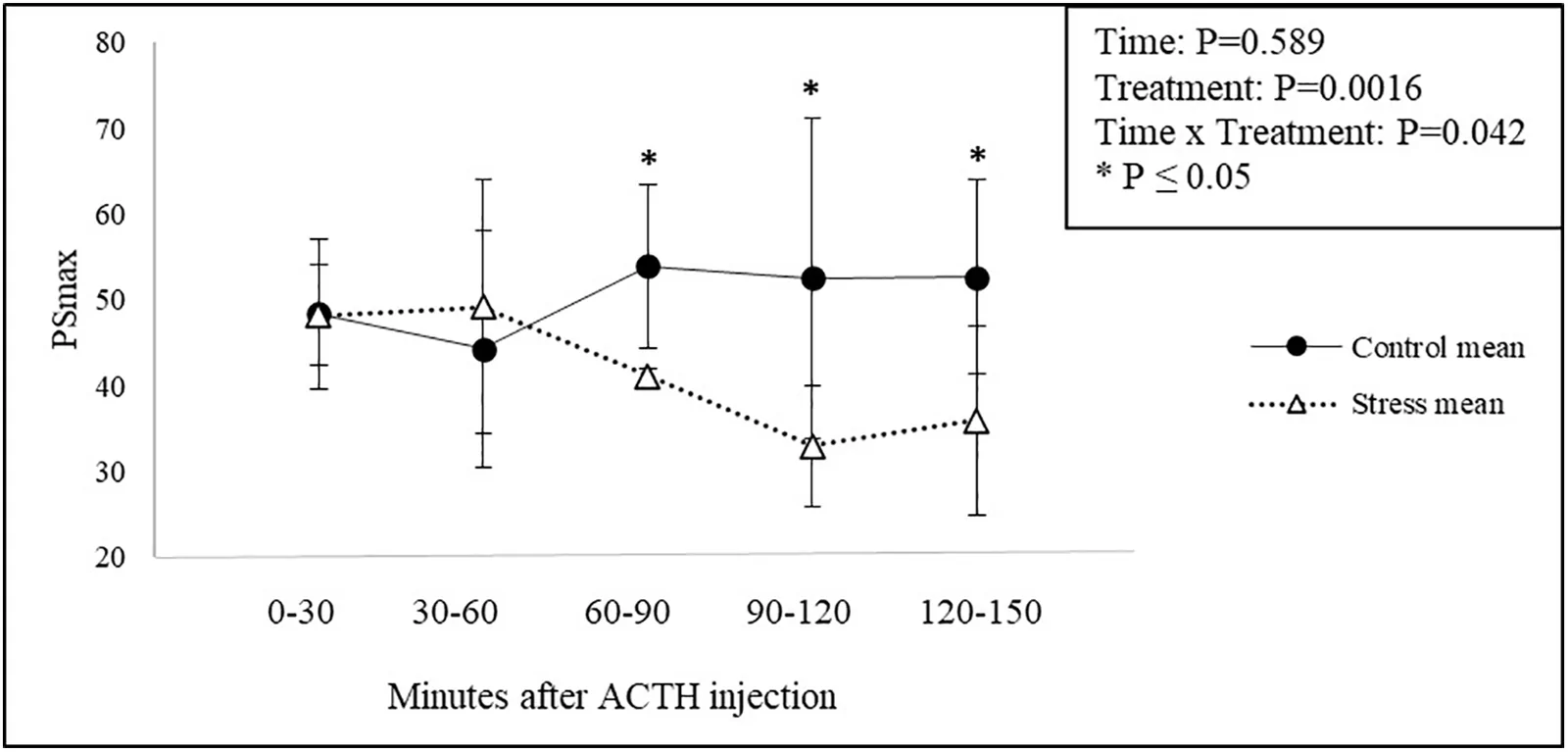
Small intestinal PSmax values (mean ± SEM) on control and ACTH-injected days, 0-150 minutes after ACTH injection.

The linear mixed-effects model demonstrated a significant main effect of Treatment (control vs. stress; P = 0.0016), while no significant main effect of Time (P = 0.589), and a significant Treatment × Time interaction (P = 0.042) on small intestinal smooth muscle activity. *Post hoc* Tukey-adjusted pairwise comparisons revealed that small intestinal activity was significantly lower under the stress condition during the later measured intervals. Specifically, significant differences between control and stress days were observed at 60–90 minutes (P = 0.036), 90–120 minutes (P = 0.002), and 120–150 minutes (P = 0.007), whereas no differences were detected during the earlier periods (0–30 and 30–60 minutes) (Fig 8.).

The delayed onset of the motility reduction corresponds well with the known physiological kinetics of ACTH-driven cortisol elevation, which typically exerts its gastrointestinal effects with some latency [10]. Previous rat studies have demonstrated that acute stress selectively suppresses small intestinal motility while leaving gastric emptying largely unaffected, indicating region-specific sensitivity of the gut to stress exposure [17]. These stress-induced alterations are mediated via centrally acting corticotropin-releasing factor-dependent neuroendocrine pathways [19]. Together, these findings support the interpretation that the reduction in small intestinal smooth muscle activity observed between 90 and 150 minutes after ACTH administration reflects downstream neuroendocrine processes rather than an immediate stress response. Thus, the current results suggest that small intestinal smooth muscle activity may serve as a more sensitive physiological marker of mild stress compared to gastric activity.

**Fig 9:**
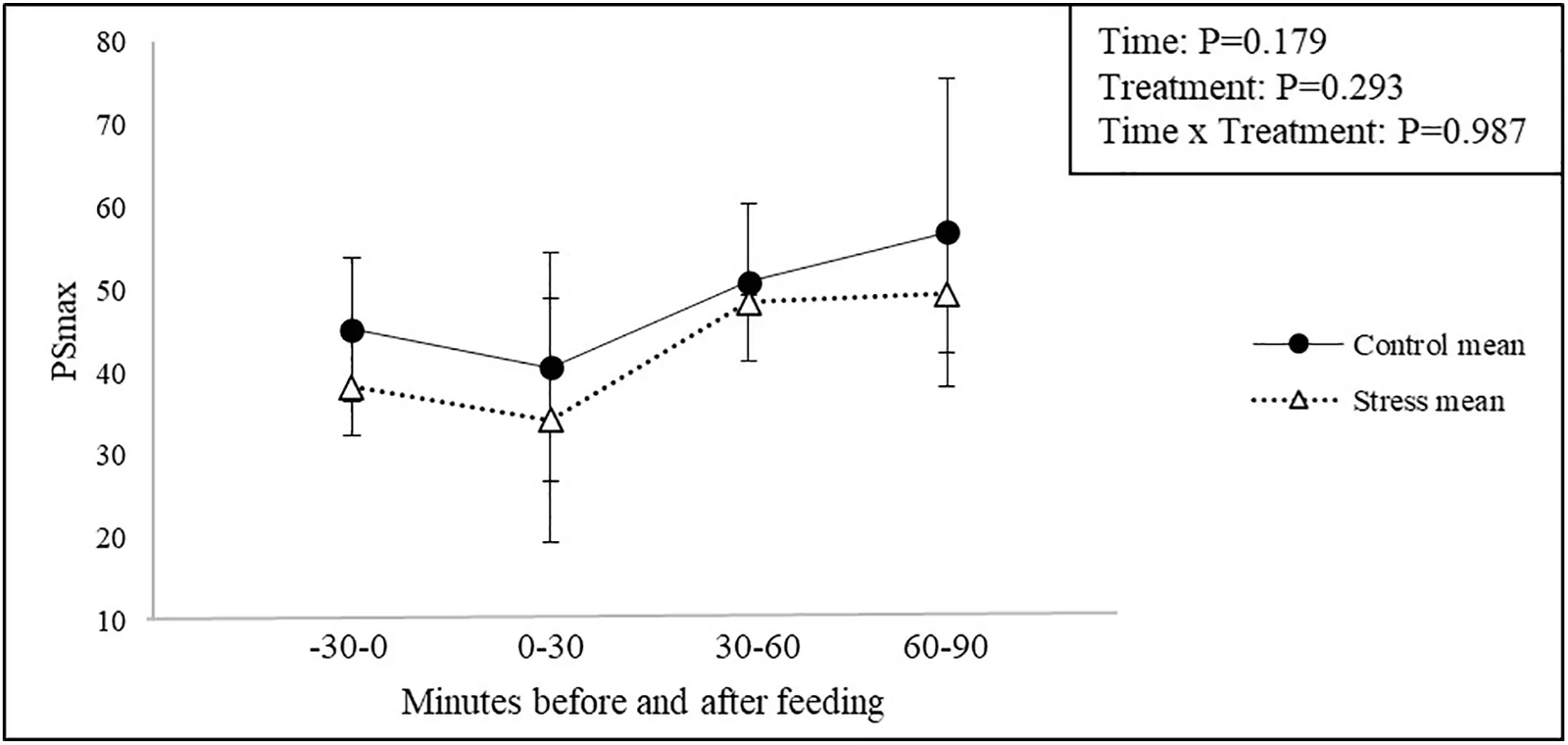
Small intestinal PSmax values (mean ± SEM) on control and ACTH-injected days, 30 minutes before and 90 minutes after feeding.

During the feeding-related observation period, in which feeding (minute 0) occurred four hours after ACTH injection, the linear mixed-effects model revealed no significant main effects of Treatment (control vs. stress; P = 0.293), Time (P = 0.179), and Treatment × Time interaction (P = 0.987) on small intestine smooth muscle activity. Mean values showed numerically lower activity values under the stress condition at all sampling intervals; however, none of these differences reached statistical significance (Fig 9.).

The hypothalamic-pituitary-adrenal (HPA) axis response to acute stimulation follows a well-defined temporal sequence. While ACTH is released rapidly in response to a stressor, the subsequent rise in circulating glucocorticoids, such as cortisol, is delayed due to the time required for adrenal synthesis and secretion. In pigs, plasma cortisol concentrations have been shown to peak approximately one hour after ACTH administration, followed by a gradual decline toward baseline levels within 2–3 hours under non-chronic conditions [10]. Consistent with this, rodent models of HPA axis dynamics demonstrate that cortisol responses to acute stress typically develop over tens of minutes and resolve over a similar 2–3-hour time frame, depending on stressor intensity and the efficiency of negative feedback regulation [20–22].The interpretation of these findings should also consider the frequency ranges applied for signal analysis. Gastrointestinal myoelectrical activity can be expressed either in hertz (Hz) or in CPM, the latter being the convention most commonly used in gastrointestinal myoelectrical studies and therefore applied in the present study. As comparable porcine reference data remain limited, previously published rat-derived frequency ranges were considered as a reference point [8]. However, considering available porcine studies and species-specific differences in gastrointestinal myoelectrical activity, the small intestinal frequency window was defined as 10–20 CPM [4,23,24], rather than the 20–25 CPM range reported in rats [8]. The gastric (3–5 CPM) and large intestinal (1–3 CPM) frequency windows corresponded to the ranges described in the original classification [8].

### Large intestinal smooth muscle activity

**Fig 10:**
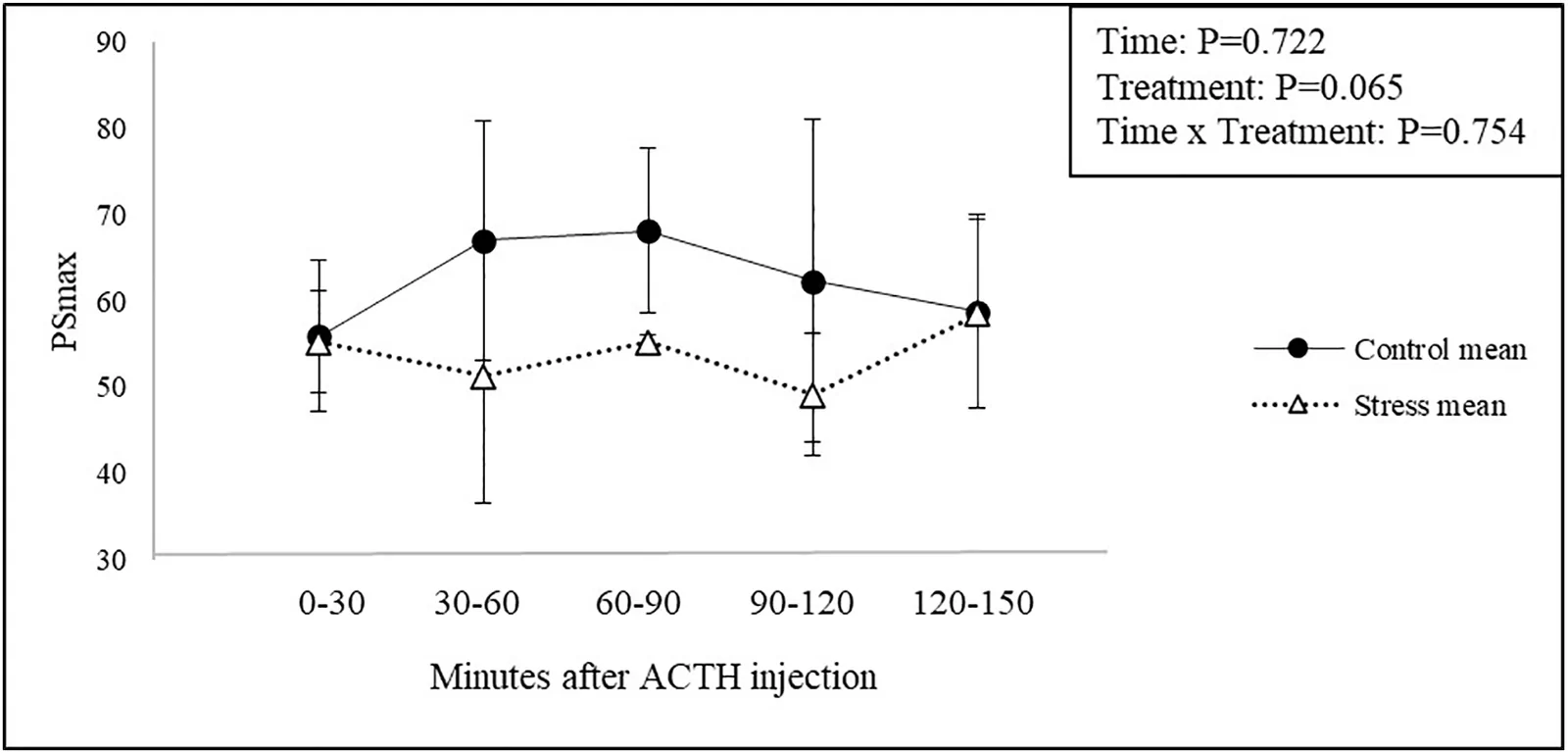
Large intestinal PSmax values (mean ± SEM) on control and ACTH-injected days, 0-150 minutes after ACTH injection.

The linear mixed-effects model revealed a trend-level main effect of Treatment (control vs. stress; P = 0.065) on large intestinal smooth muscle activity, indicating overall lower activity under the stress condition compared with the control condition. No significant main effect of Time was detected (P = 0.722), and no significant Treatment × Time interaction was observed (P = 0.754) (Fig 10.).

Stress-induced alterations in gastrointestinal motility, including reduced small intestinal transit and increased colonic activity, have been shown to be mediated by endogenous corticotropin-releasing factor (CRF) in rats [19]. Psychological stress has been shown to selectively modulate colonic motility in humans, with patients with irritable bowel syndrome exhibiting significant increases during stress exposure and subsequent decreases afterward, whereas healthy controls showed no significant changes [25]. In contrast, the current study in pigs did not confirm a statistically significant increase in large intestinal smooth muscle activity under the applied mild ACTH-induced stress protocol. This discrepancy may be partly explained by the fact that, in pigs, the characteristic CPM for each gastrointestinal segment have not been precisely established, which limits the ability to filter and interpret SMEMG signals accurately; in our analysis, we relied on reference CPM values obtained from rat models [8], whose applicability and physiological relevance in pigs have been experimentally validated [3].

**Fig 11:**
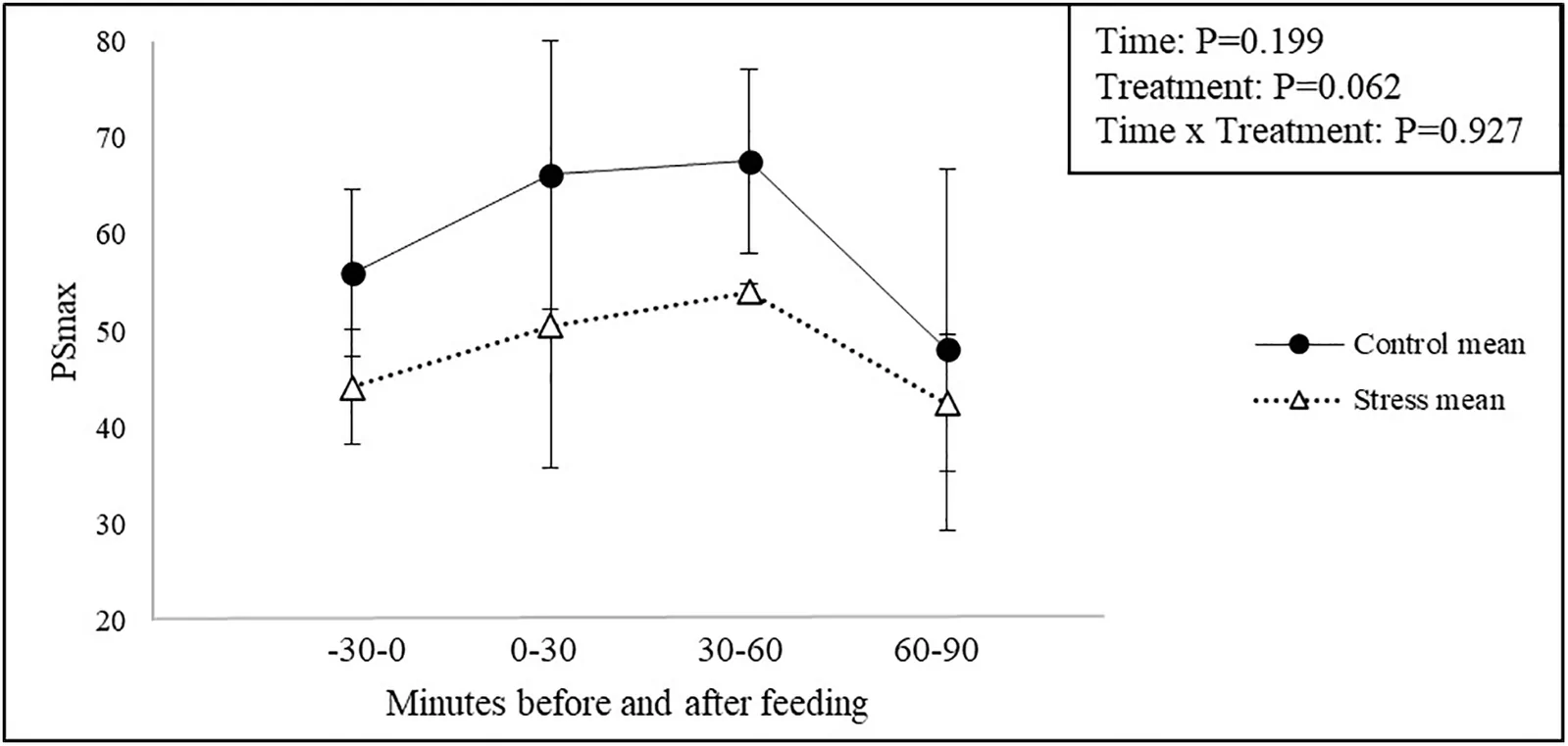
Large intestinal PSmax values (mean ± SEM) on control and ACTH-injected days, 30 minutes before and 90 minutes after feeding.

During the feeding period, with feeding (minute 0) occurring four hours after ACTH injection, large intestinal smooth muscle activity tended to be lower on the stress-induced day compared with control during the feeding period (P = 0.062). Motility did not significantly vary across time points (P = 0.199), and no Treatment × Time interaction was observed (P = 0.927), indicating that the temporal motility pattern was similar in both groups. Despite the lack of significant effects, mean values showed reduced contractile activity on the stress day in the feeding-associated time window, indicating a consistent downward shift in contractile activity (Fig 11.).

Although large intestinal smooth muscle activity did not differ significantly between control and stress conditions during the feeding period, estimated marginal means consistently indicated lower activity under stress across all feeding-related time points. This trend suggests a subtle stress-related modulation of colonic motility that not reached statistical significance due to the mild nature of the ACTH-induced stress and the limited sample size. In contrast to several studies reporting stress-induced increases in colonic motility-primarily in rodent models or in human patients exposed to psychological or restraint stress [19,25] the present findings indicate a tendency toward reduced colonic activity in habituated pigs under endocrine-driven stress conditions.

In pigs, segmental colonic motility is influenced by complex neuroendocrine and enteric regulatory mechanisms, which may result in more subtle and variable responses to mild endocrine stressors. Previous porcine *in vivo* studies have demonstrated that gastrointestinal myoelectrical activity can be assessed using CPM-based approaches, yielding physiologically coherent segmental patterns [3,4]. However, it should be noted that CPM reference ranges are not strictly interchangeable across species and gastrointestinal segments. In the present study, rat-derived reference values applied for gastric and small intestinal activity were consistent with previously reported physiological frequency bands, whereas notable differences were observed in colonic CPM ranges. Specifically, rodent-derived values (1–3 CPM), obtained under *ex vivo* organ preparation conditions [8], differ from *in vivo* porcine measurements reported by Salimi-Jazi et al. [4], where colonic activity is typically centered around 5–6 CPM, reflecting intact neuroenteric regulation. Importantly, this discrepancy appears to be largely confined to the large intestine, while gastric and small intestinal CPM ranges remain broadly comparable across species and experimental conditions. In this context, the observed tendency toward reduced large intestinal activity under stress may represent a mild modulatory effect of ACTH-induced endocrine activation, which did not reach statistical significance. Taken together, these findings suggest that while porcine colonic myoelectrical activity exhibits a trend toward reduced activity under acute ACTH-induced stress, the use of rat-derived CPM reference thresholds obtained under *ex vivo* conditions may have selectively affected colonic sensitivity in cross-species comparison, potentially contributing to the absence of statistically significant differences between experimental conditions.

## Conclusions

The present findings indicate that SMEMG may serve as a promising non-invasive method for detecting stress-induced alterations in gastrointestinal motility in pigs. Statistically significant reductions were observed in small intestinal smooth muscle activity. Large intestinal measurements showed a consistent trend toward decreased activity under stress. Collectively, the pattern suggests that SMEMG is sufficiently sensitive to detect stress-related reductions across multiple segments of the digestive tract and may hold future potential as a tool for stress monitoring. Validation in larger cohorts and under diverse stress paradigms will be essential to determine the robustness and generalizability of this approach.

## References

[1] Világi I. Neurokémia. Budapest–Pécs: Dialóg Campus Kiadó; 2003.

[2] Sanders KM, Koh SD, Ward SM. Interstitial cells of cajal as pacemakers in the gastrointestinal tract. Annu Rev Physiol. 2006;68:307–43. doi: 10.1146/annurev.physiol.68.040504.094718 16460275

[3] Nagy K, Fébel H, Bazar G, Grosz G, Gáspár R, Ferenc Szűcs K, et al. Non-invasive smooth muscle electromyography (SMEMG) as a novel monitoring technology of the gastrointestinal tract of awake, free-moving pigs-A pilot study. PLoS One. 2021;16(9):e0257311. doi: 10.1371/journal.pone.0257311 34516588 PMC8437306

[4] Salimi-Jazi F, Thomas AL, Rafeeqi T, Diyaolu M, Wood LSY, Axelrod S, et al. Gastrointestinal myoelectric measurements via simultaneous external and internal electrodes in pigs. J Surg Res. 2022;279:119–26. doi: 10.1016/j.jss.2022.05.012 35759929

[5] Nakade Y, Tsuchida D, Fukuda H, Iwa M, Pappas TN, Takahashi T. Restraint stress augments postprandial gastric contractions but impairs antropyloric coordination in conscious rats. Am J Physiol Regul Integr Comp Physiol. 2006;290(3):R616–24. doi: 10.1152/ajpregu.00161.2005 16254129

[6] Plourde V. Stress-induced changes in the gastrointestinal motor system. Can J Gastroenterol. 1999;13 Suppl A:26A–31A. doi: 10.1155/1999/320626 10202205

[7] Gue M, Fioramonti J, Bueno L. Comparative influences of acoustic and cold stress on gastrointestinal transit in mice. Am J Physiol. 1987;253(2 Pt 1):G124–8. doi: 10.1152/ajpgi.1987.253.2.G124 3497584

[8] Szucs KF, Nagy A, Grosz G, Tiszai Z, Gaspar R. Correlation between slow-wave myoelectric signals and mechanical contractions in the gastrointestinal tract: Advanced electromyographic method in rats. J Pharmacol Toxicol Methods. 2016;82:37–44. doi: 10.1016/j.vascn.2016.07.005 27475721

[9] Plut J, Snoj T, Golinar Oven I, Štukelj M. The Combination of Serum and Oral Fluid Cortisol Levels and Welfare Quality Protocol® for Assessment of Pig Welfare on Intensive Farms. Agriculture. 2023;13(2):351. doi: 10.3390/agriculture13020351

[10] Madej A, Mwanza AM, Kindahl H, Einarsson S. Effect of ACTH and CRH on plasma levels of cortisol and prostaglandin F2alpha metabolite in cycling gilts and castrated boars. Acta Vet Scand. 2005;46(4):249–56. doi: 10.1186/1751-0147-46-249 16398336 PMC1618968

[11] Janssens CJ. Chronic stress and pituitary-adrenal function in female pigs [Doctoral dissertation]. Wageningen University & Research; 1994. Available from: https://edepot.wur.nl/207067

[12] Geverink NA, Schouten WGP, Gort G, Wiegant VM. Individual differences in behavioral and physiological responses to restraint stress in pigs. Physiol Behav. 2002;77(2–3):451–7. doi: 10.1016/s0031-9384(02)00877-6 12419422

[13] Lopez-Duran NL, Hajal NJ, Olson SL, Felt BT, Vazquez DM. Individual differences in cortisol responses to fear and frustration during middle childhood. J Exp Child Psychol. 2009;103(3):285–95. doi: 10.1016/j.jecp.2009.03.008 19410263 PMC2705941

[14] Schlotz W, Hammerfald K, Ehlert U, Gaab J. Individual differences in the cortisol response to stress in young healthy men: testing the roles of perceived stress reactivity and threat appraisal using multiphase latent growth curve modeling. Biol Psychol. 2011;87(2):257–64. doi: 10.1016/j.biopsycho.2011.03.005 21419825

[15] Selye H. The physiology and pathology of exposure to stress. Montreal: Acta Inc. Medical Publishers. 1950.

[16] Herman JP, McKlveen JM, Ghosal S, Kopp B, Wulsin A, Makinson R, et al. Regulation of the Hypothalamic-Pituitary-Adrenocortical Stress Response. Compr Physiol. 2016;6(2):603–21. doi: 10.1002/cphy.c150015 27065163 PMC4867107

[17] Williams CL, Villar RG, Peterson JM, Burks TF. Stress-induced changes in intestinal transit in the rat: a model for irritable bowel syndrome. Gastroenterology. 1988;94(3):611–21. doi: 10.1016/0016-5085(88)90231-4 2828144

[18] Zheng J, Dobner A, Babygirija R, Ludwig K, Takahashi T. Effects of repeated restraint stress on gastric motility in rats. Am J Physiol Regul Integr Comp Physiol. 2009;296(5):R1358–65. doi: 10.1152/ajpregu.90928.2008 19261914

[19] Lenz HJ, Raedler A, Greten H, Vale WW, Rivier JE. Stress-induced gastrointestinal secretory and motor responses in rats are mediated by endogenous corticotropin-releasing factor. Gastroenterology. 1988;95(6):1510–7. doi: 10.1016/s0016-5085(88)80070-2 2846402

[20] Solomon MB, Loftspring M, de Kloet AD, Ghosal S, Jankord R, Flak JN, et al. Neuroendocrine Function After Hypothalamic Depletion of Glucocorticoid Receptors in Male and Female Mice. Endocrinology. 2015;156(8):2843–53. doi: 10.1210/en.2015-1276 26046806 PMC4511133

[21] Vahl TP, Ulrich-Lai YM, Ostrander MM, Dolgas CM, Elfers EE, Seeley RJ, et al. Comparative analysis of ACTH and corticosterone sampling methods in rats. Am J Physiol Endocrinol Metab. 2005;289(5):E823–8. doi: 10.1152/ajpendo.00122.2005 15956051

[22] Choi DC, Furay AR, Evanson NK, Ostrander MM, Ulrich-Lai YM, Herman JP. Bed nucleus of the stria terminalis subregions differentially regulate hypothalamic-pituitary-adrenal axis activity: implications for the integration of limbic inputs. J Neurosci. 2007;27(8):2025–34. doi: 10.1523/JNEUROSCI.4301-06.2007 17314298 PMC6673539

[23] Hudson NPH, Mayhew IG, Pearson GT. Interstitial cells of Cajal and electrical activity of smooth muscle in porcine ileum. Acta Physiol (Oxf). 2006;187(3):391–7. doi: 10.1111/j.1748-1716.2006.01589.x 16776664

[24] Groner JI, Altschuler SM, Ziegler MM. The newborn piglet: a model of neonatal gastrointestinal motility. J Pediatr Surg. 1990;25(3):315–8. doi: 10.1016/0022-3468(90)90075-k 2313500

[25] Fukudo S, Suzuki J. Colonic motility, autonomic function, and gastrointestinal hormones under psychological stress on irritable bowel syndrome. Tohoku J Exp Med. 1987;151(4):373–85. doi: 10.1620/tjem.151.373 3617051

