# Supplementary material for: Surface electromyography as a non-invasive method for detecting stress-induced changes in gastrointestinal motility in pigs
Source: PLoS One. 2026 Sep 15;21(9):e0358481. doi: 10.1371/journal.pone.0358481 (PMC13577549; doi:10.1371/journal.pone.0358481)
Supplement: Supplementary file 1 [file pone.0358481.s002.pdf]

Response to Reviewers

[PONE-D-26-21675]
Surface Electromyography as a Non-Invasive Method for Detecting Stress-Induced Changes in Gastrointestinal Motility in Pigs

The authors would like to thank both the Editor and the Reviewer for considering the manuscript. All comments and recommendations have been thankfully accepted and corrections have been made accordingly. The changes are highlighted with Track Changes in the revised version of the manuscript. Please find our specific replies below.

Journal Requirements:

1. „Please ensure that your manuscript meets PLOS ONE's style requirements, including those for file naming."

- The requirements were reviewed again, and all necessary modifications were implemented.

2. „To comply with PLOS One submissions requirements, in your Methods section, please provide additional information regarding the experiments involving animals and ensure you have included details on (1) methods of sacrifice, (2) methods of anesthesia and/or analgesia, and (3) efforts to alleviate suffering."

- The manuscript was expanded in L101-104 and L115-127.
    - L101-104: "At the end of the study, the animals were not euthanized. Following completion of the experimental procedures, all pigs underwent veterinary examination, and after confirmation of their healthy clinical status, they were returned to the food production chain in accordance with the approved animal welfare protocol."
    - L115-127: "To enable repeated blood sampling (16 samples/day) and intravenous ACTH administration while minimizing procedure-related stress, permanent jugular venous catheters were surgically implanted 4 days before the experiment. General anesthesia was induced with intramuscular Zoletil® 50 (4 mg/kg), followed by inhalational anesthesia with isoflurane in oxygen. After induction, the animals were intubated, and anesthesia was maintained with 2% isoflurane in oxygen. The catheter was inserted into the jugular vein through a small skin incision and secured with sutures. Local analgesia was provided by infiltration and topical application of 1% lidocaine prior to intubation and venous catheterization. Physiological parameters, including electrocardiogram (ECG), oxygen saturation, and body temperature, were continuously monitored throughout surgery. Following surgery, the incision site and sutures were inspected daily and cleaned as needed throughout the experimental period. Catheter patency was maintained by daily flushing with heparinized saline. All

procedures were performed by appropriately trained personnel in accordance with the approved animal welfare protocol."

3. „Please note that PLOS One has specific guidelines on code sharing for submissions in which author-generated code underpins the findings in the manuscript. In these cases, we expect all author-generated code to be made available without restrictions upon publication of the work."

- The R code used for the statistical analyses was deposited in Zenodo and is available at the following link: https://doi.org/10.5281/zenodo.21720478
- The following sentence was added to the manuscript at L196-197:
„The R scripts used for statistical analyses are publicly available at https://doi.org/10.5281/zenodo.21720478."

4. The Funding Statement was included with the following statement: "There was no additional external funding received for this study."

5. The financial disclosure was included with the following statement: „The funders had no role in study design, data collection and analysis, decision to publish, or preparation of the manuscript."

6. The separate captions for each figure were added into the manuscript.

7. The reviewer comments did not recommend to cite specific previously published works.

8. The reference list was reviewed and corrected where necessary.

Reviewers' comments:

We sincerely thank the reviewer for the positive evaluation of our work and for recognizing the novelty of applying surface electromyography to assess gastrointestinal smooth muscle activity. We fully agree that further studies involving a larger number of animals are needed to validate and strengthen the present findings.

At the same time, the present study was designed in accordance with the principles of the 3Rs (Replacement, Reduction, and Refinement) and animal welfare considerations. Since this was the first study investigating whether surface electromyography could reliably detect stress-induced changes in gastrointestinal motility under these experimental conditions, we deliberately used the minimum number of animals necessary to evaluate the feasibility of the method. Furthermore, each sEMG measurement required extensive preparation and individual handling of the animals, making the experiments particularly labor-intensive. Having demonstrated the feasibility of the approach, we plan to extend our investigations to a larger number of animals in future studies.

We also agree that the non-invasive nature of the sEMG technique offers promising opportunities for investigating gastrointestinal motility and the factors influencing it. We believe that this approach has considerable potential for future applications in animal nutrition and feeding research, where repeated, non-invasive measurements are particularly advantageous.

Answers to 5/1-4:

We sincerely thank again the reviewer for pointing out this important details. The modifications have been made to all points and are added to the revised manuscript.

1. What electrodes were used, and how were they attached to the skin?

- The additional information was added to the manuscript in L148-150:

  „ SMEMG data were collected using a Holter device (DR4CH01, MSB-Met Kft., Hungary) connected to two disposable, self-adhesive foam surface electrodes (3M™ Red Dot™, 4 × 3.3 cm; 3M, St. Paul, USA) placed after shaving the area and cleaning the skin with 96% (v/v) ethanol."

2. It should be clarified whether the electrical signals were amplified using an amplifier and whether filters were used to reduce artifacts?

- The additional information was added to the manuscript in L159-163:

  „The electrical signals were recorded using the Holter recording system, which included built-in hardware signal conditioning. The gastrointestinal electrical activity channels were amplified with a gain of ×2000 and recorded with a bandwidth of 0.01–1 Hz. Signals were digitized at a sampling rate of 1000 samples/s (1000 Hz). No additional preprocessing or artifact-reduction procedures were applied by the Holter system prior to signal analysis."

3. Please explain what is CMP in the text of chapter (L148-151). This information is in ''Intoduction'' (L 72), but in the description of the methods this information should also be included.

- The additional information was added to the manuscript in L168-170:

  „The frequency unit CPM is conventionally used for the characterization of gastrointestinal myoelectrical activity; for comparison with SI frequency units, CPM values can be converted to Hertz (Hz; 1 Hz = 60 cycles/min)."

4. Please explain what is PsDmax in this place (or explain in the description of each figure). This parameter is then indicated in the figures.

- The additional information was added to the manuscript in L174-179:

  „ Following frequency filtering, Fast Fourier Transform (FFT) analysis was performed within each predefined organ-specific frequency range, and the Power Spectrum maximum (PSmax) value was determined for each 30-minute interval. PSmax was defined as the maximum value of the power spectrum curve obtained from FFT analysis, representing the highest spectral power detected within the selected frequency range. Gastrointestinal myoelectrical activity was quantified based on these PSmax values."

5. The Authors assumed CPM of 3-5, 10-20, and 1-3 for the stomach, small intestine, and large intestine, respectively, referring to the research of Szűcs et al. (L151). In the above-mentioned work, the myoelectric cycle frequency of the ileum was 20-25. Please clarify.

- The additional information was added to the manuscript in L305-314:

„ The interpretation of these findings should also consider the frequency ranges applied for signal analysis. Gastrointestinal myoelectrical activity can be expressed either in hertz (Hz) or in CPM, the latter being the convention most commonly used in gastrointestinal myoelectrical studies and therefore applied in the present study. As comparable porcine reference data remain limited, previously published rat-derived frequency ranges were considered as a reference point [8]. However, considering available porcine studies and species-specific differences in gastrointestinal myoelectrical activity, the small intestinal frequency window was defined as 10–20 CPM [4, 23, 24], rather than the 20–25 CPM range reported in rats [8]. The gastric (3–5 CPM) and large intestinal (1–3 CPM) frequency windows corresponded to the ranges described in the original classification [8]."

6. In chapter ''Results&Dissucion" the Authors could present the record primary myoelectric signal of the GIT. The record of specific spectra from FFT analysis for the stomach, small, and large intestines must be presented. The CPM assumptions for individual segments of GIT will then be confirmed.

- The primary gastrointestinal myoelectric signals are recorded and displayed by the software as continuous waveforms. As these recordings consist of continuous time-domain waveforms acquired over 8 hours, we considered that presenting the complete raw recordings for all animals would be impractical within the scope of the manuscript. Therefore a representative example of the primary myoelectric recording has now been included in the revised manuscript, while screenshots illustrating the software workflow and FFT settings are provided below for further clarification. The software processes the recorded time-domain signals using FFT analysis and directly exports the resulting numerical parameters in tabular format. However, the software does not provide exportable graphical frequency spectra suitable for publication. Therefore, the quantitative FFT-derived numerical data used for all statistical analyses have been deposited in Zenodo and are publicly available at https://doi.org/10.5281/zenodo.19951958. All figures presented in the "Results&Discussion" section of the revised manuscript were generated from these FFT-derived numerical data. The primary gastrointestinal myoelectric signals are recorded and displayed by the software as continuous waveforms. Because these recordings consist of continuous time-domain waveforms acquired over 8 hours, presenting the complete raw recordings for all animals within the manuscript would not be practical.

The raw myoelectric recordings are visualized in the software as continuous waveforms rather than numerical data:

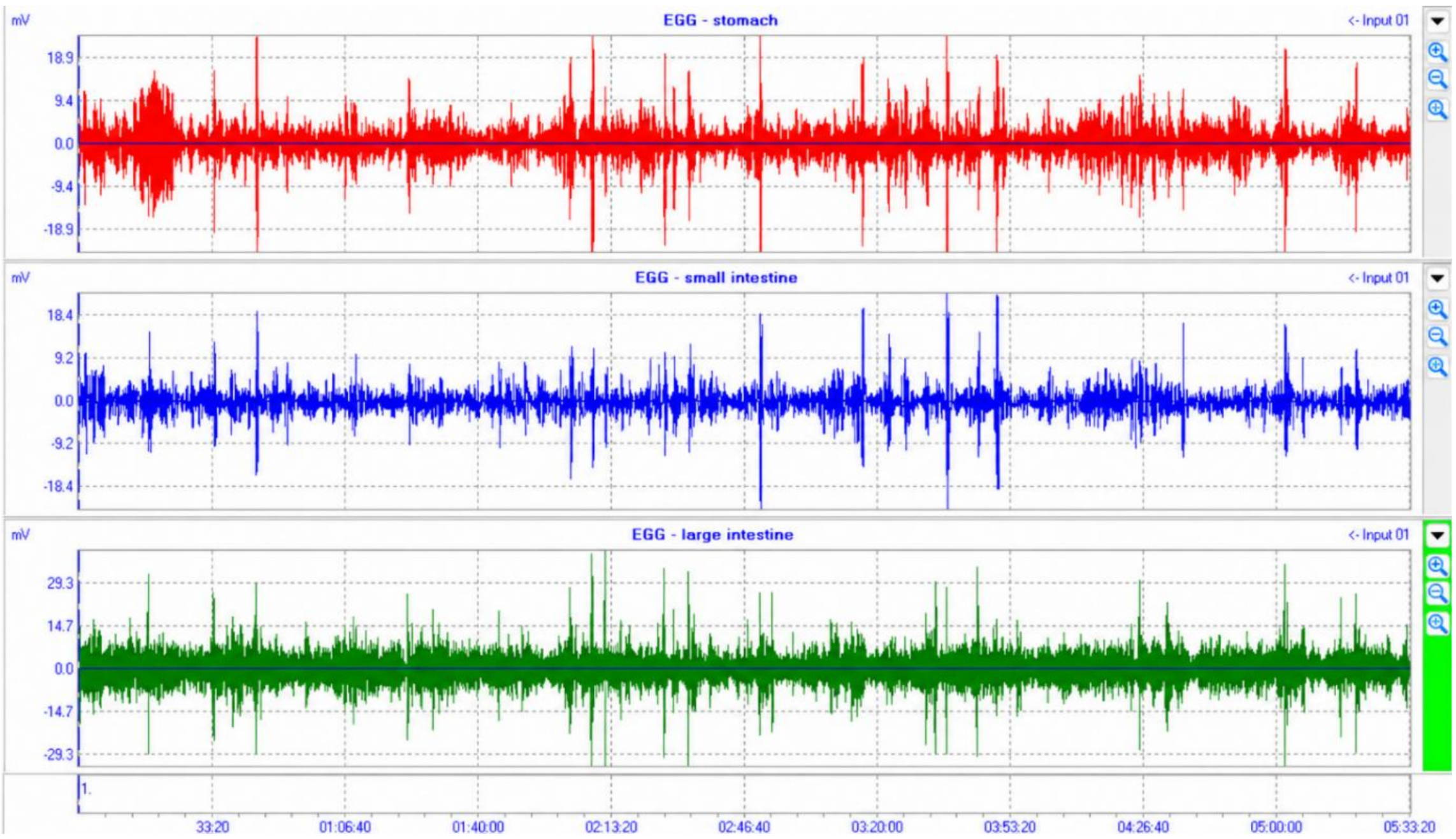

The time window displayed by the software can be adjusted according to the desired level of temporal resolution. In the previous screenshot, the maximum time interval interval that can be displayed simultaneously in the waveform view is shown (5 h 30 min), whereas the following figure shows a 1 min 50 s time window (the x-axis shows the seconds), in which the recorded waveforms can be visualized in greater detail. When the display is sufficiently magnified, the software provides the recorded voltage (mV) values at individual time points; however, these absolute voltage values alone are not the primary parameter of interest for the assessment of gastrointestinal smooth muscle activity. Rather, the relevant information is contained in the temporal changes of the electrical potential, which reflect the underlying myoelectric activity. The quantitative parameters used for the analyses were obtained following FFT processing and have been deposited in Zenodo, as described above, and are fully publicly available through the provided DOI.

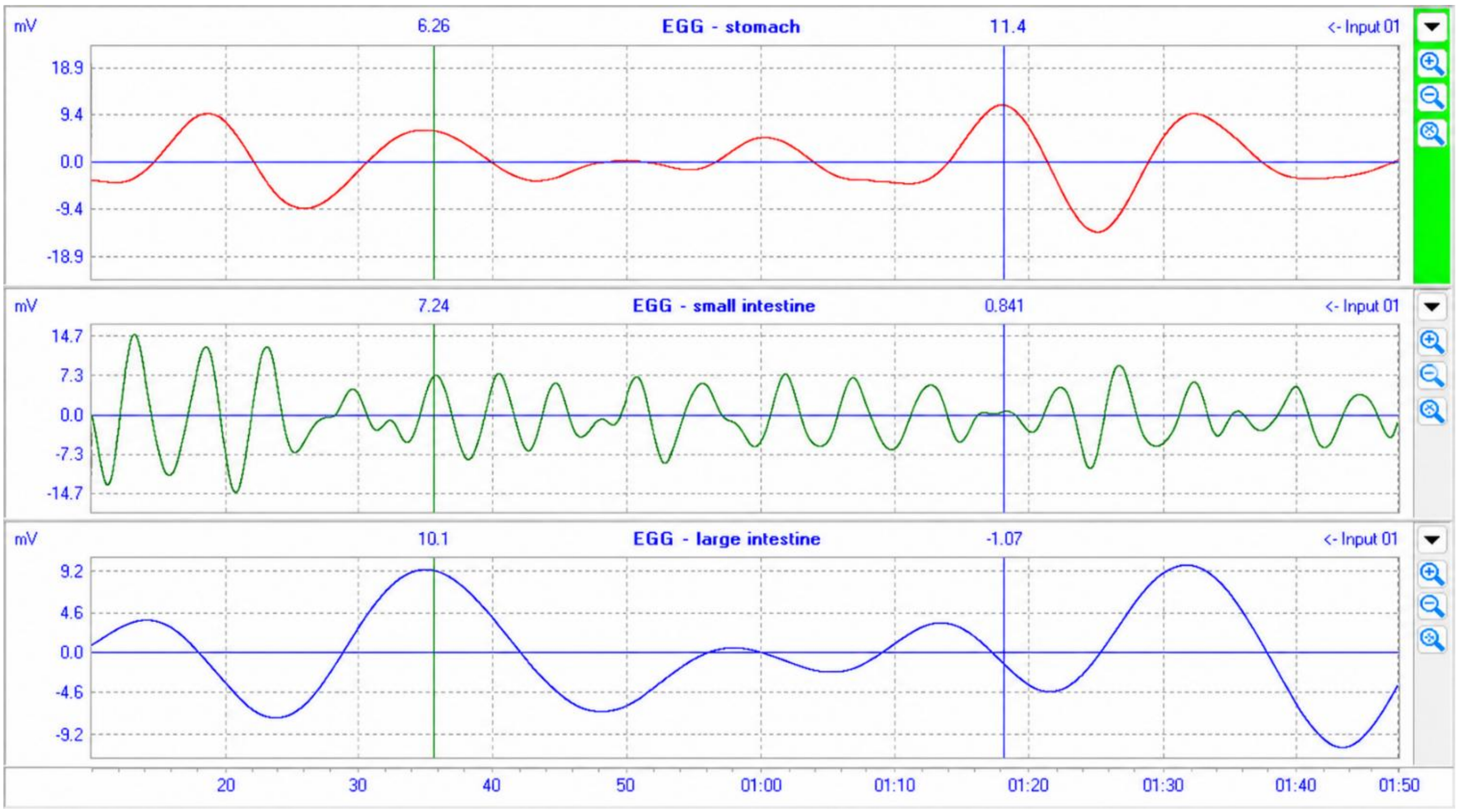

The following screenshot shows the software settings used to perform FFT every 30 minutes for the selected gastrointestinal segments to calculate the PSmax values:

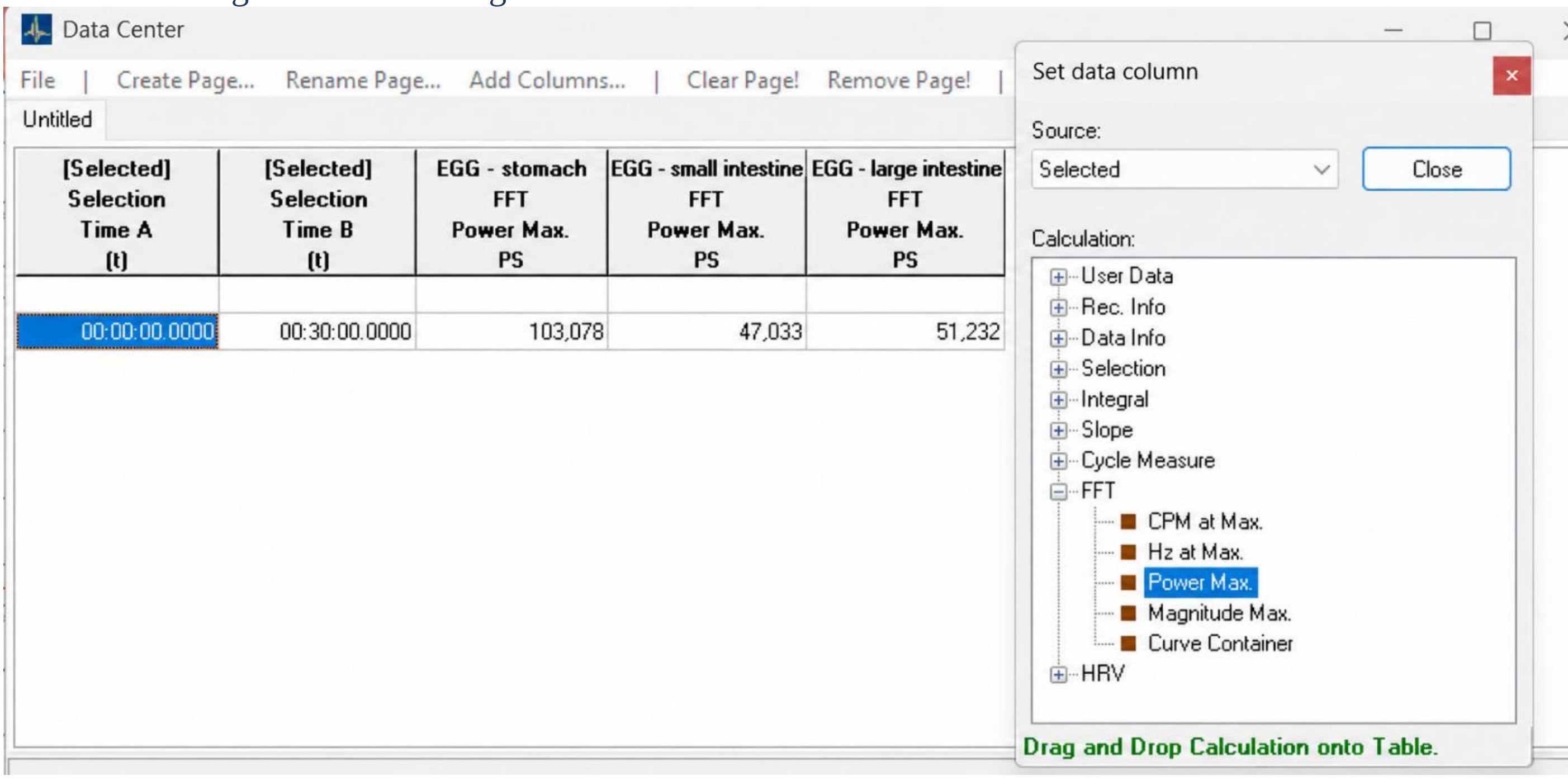

Representative screenshots from the software are provided to illustrate the raw recordings and the corresponding CPM values used for the selected gastrointestinal segments:

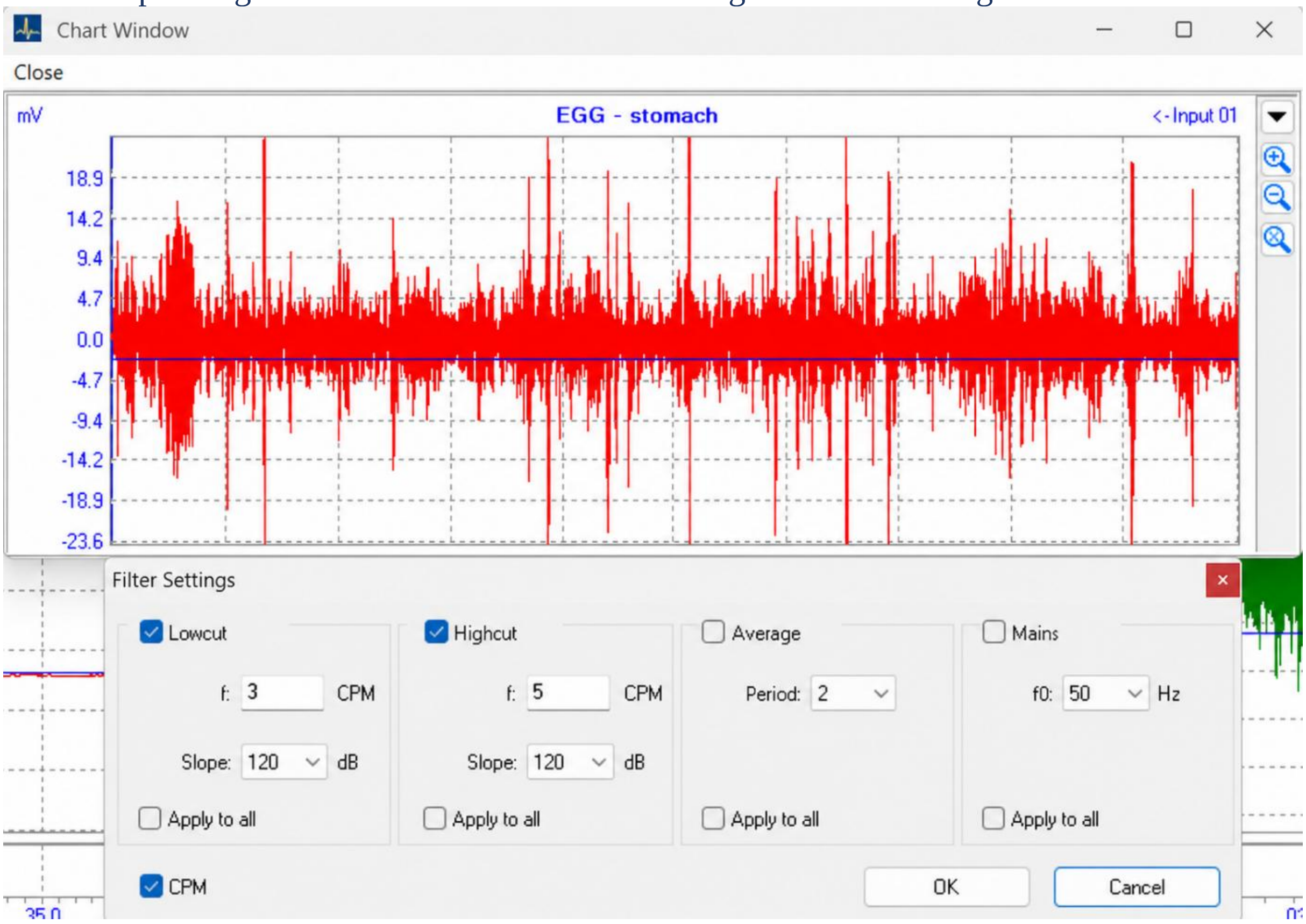

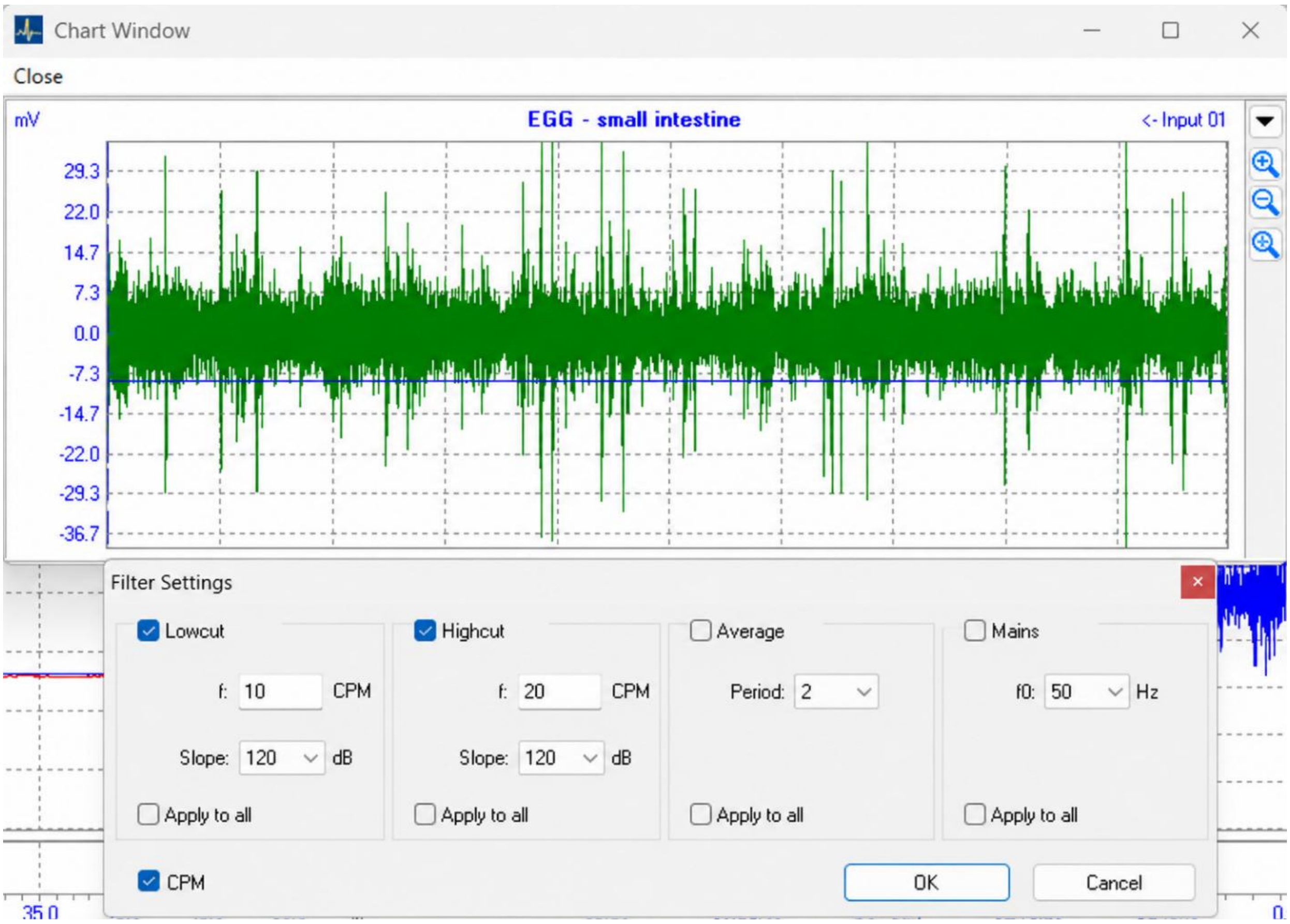
Chart Window
Close
mV
EGG - small intestine
<- Input 01
29.3
22.0
14.7
7.3
0.0
-7.3
-14.7
-22.0
-29.3
-36.7
Filter Settings
Lowcut
f: 10 CPM
Slope: 120 dB
Apply to all
Highcut
f: 20 CPM
Slope: 120 dB
Apply to all
Average
Period: 2
Apply to all
Mains
f0: 50 Hz
Apply to all
CPM
OK
Cancel

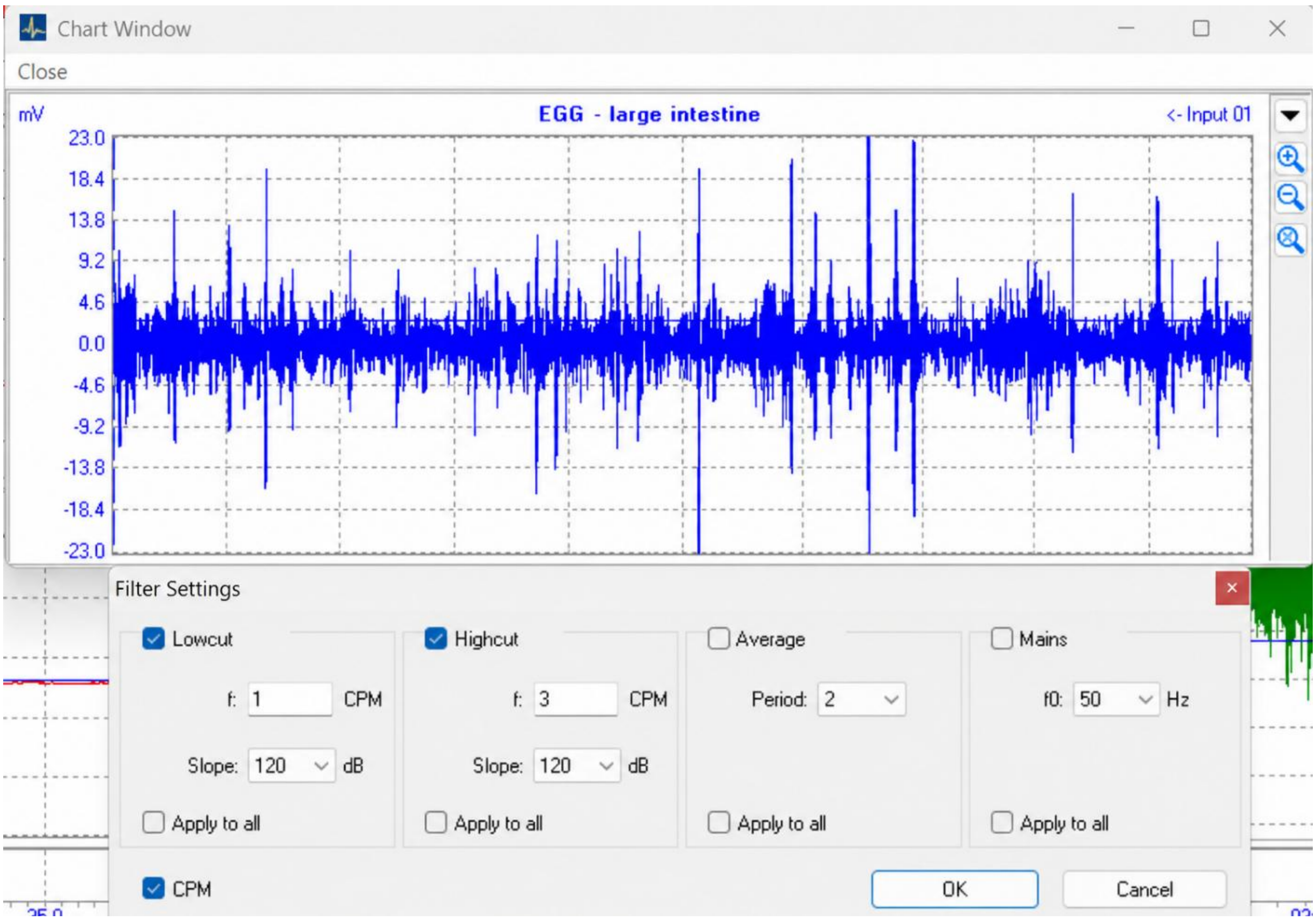
Chart Window
Close
mV
EGG - large intestine
<- Input 01
23.0
18.4
13.8
9.2
4.6
0.0
-4.6
-9.2
-13.8
-18.4
-23.0
Filter Settings
Lowcut
f: 1 CPM
Slope: 120 dB
Apply to all
Highcut
f: 3 CPM
Slope: 120 dB
Apply to all
Average
Period: 2
Apply to all
Mains
f0: 50 Hz
Apply to all
CPM
OK
Cancel

We sincerely thank the Editor and the Reviewer for their constructive comments and valuable suggestions, which have helped us improve the quality and clarity of the manuscript. We hope that the revised manuscript satisfactorily addresses all comments and thank you for your consideration.

Sincerely,
Orsolya Csötönyi
Corresponding Author, on behalf of all authors
